# The Compound U18666A Inhibits the Intoxication of Cells by *Clostridioides difficile* Toxins TcdA and TcdB

**DOI:** 10.3389/fmicb.2021.784856

**Published:** 2021-11-29

**Authors:** Panagiotis Papatheodorou, Selina Kindig, Adriana Badilla-Lobo, Stephan Fischer, Ebru Durgun, Tharani Thuraisingam, Alexander Witte, Shuo Song, Klaus Aktories, Esteban Chaves-Olarte, César Rodríguez, Holger Barth

**Affiliations:** ^1^Institute of Pharmacology and Toxicology, Ulm University Medical Center, Ulm, Germany; ^2^Centro de Investigación en Enfermedades Tropicales and Facultad de Microbiología, Universidad de Costa Rica, San José, Costa Rica; ^3^Institute of Experimental and Clinical Pharmacology and Toxicology, Albert Ludwig University Freiburg, Freiburg, Germany

**Keywords:** bacterial toxin, toxin inhibitor, cholesterol biosynthesis, cholesterol transport, cholesterol

## Abstract

The intestinal pathogen *Clostridioides (C.) difficile* is a major cause of diarrhea both in hospitals and outpatient in industrialized countries. This bacterium produces two large exotoxins, toxin A (TcdA) and toxin B (TcdB), which are directly responsible for the onset of clinical symptoms of *C. difficile*-associated diseases (CDADs), such as antibiotics-associated diarrhea and the severe, life-threatening pseudomembranous colitis. Both toxins are multidomain proteins and taken up into host eukaryotic cells via receptor-mediated endocytosis. Within the cell, TcdA and TcdB inactivate Rho and/or Ras protein family members by glucosylation, which eventually results in cell death. The cytotoxic mode of action of the toxins is the main reason for the disease. Thus, compounds capable of inhibiting the cellular uptake and/or mode-of-action of both toxins are of high therapeutic interest. Recently, we found that the sterol regulatory element-binding protein 2 (SREBP-2) pathway, which regulates cholesterol content in membranes, is crucial for the intoxication of cells by TcdA and TcdB. Furthermore, it has been shown that membrane cholesterol is required for TcdA- as well as TcdB-mediated pore formation in endosomal membranes, which is a key step during the translocation of the glucosyltransferase domain of both toxins from endocytic vesicles into the cytosol of host cells. In the current study, we demonstrate that intoxication by TcdA and TcdB is diminished in cultured cells preincubated with the compound U18666A, an established inhibitor of cholesterol biosynthesis and/or intracellular transport. U18666A-pretreated cells were also less sensitive against TcdA and TcdB variants from the epidemic NAP1/027 *C. difficile* strain. Our study corroborates the crucial role of membrane cholesterol for cell entry of TcdA and TcdB, thus providing a valuable basis for the development of novel antitoxin strategies in the context of CDADs.

## Introduction

*Clostridioides difficile* is a (nosocomial) pathogen of the human gut and the major cause of antibiotics-associated diarrhea and pseudomembranous colitis. This bacterium produces two exotoxins, toxin A (TcdA) and toxin B (TcdB), that belong to the family of the clostridial glucosylating toxins (CGTs), also referred to as large clostridial cytotoxins (LCCs). The onset of clinical symptoms after *C. difficile* infections (CDIs) is strictly dependent on the toxins’ actions on target cells in the human gut (Kelly and LaMont, 2008; Aktories et al., 2017; Chandrasekaran and Lacy, 2017).

TcdA and TcdB are glucosylating toxins that specifically modify host cell target proteins by covalent attachment of a glucose moiety (Just et al., 1995). Both toxins utilize UDP-glucose for the mono-O-glucosylation of GTPases from the Rho and/or Ras family, which renders them inactive. Rho family members are master regulators of the actin cytoskeleton and numerous other cellular processes (Hall, 1994). Consequently, the intoxication of cultured mammalian cells with TcdA or TcdB leads amongst other effects to cell rounding due to the destruction of the actin cytoskeleton (Aktories and Just, 2005).

In order to reach their target proteins in the host cell cytosol, TcdA and TcdB need to enter cells via receptor-mediated, clathrin-dependent endocytosis (Papatheodorou et al., 2010). Both toxins are single-chain, multidomain toxins, which bind to host cell surface receptors via at least two independent, C-terminally located receptor-binding domains (Gerhard, 2017; Papatheodorou et al., 2018). After internalization of the toxin/receptor complexes, the pH of the endosomal lumen decreases, triggering membrane insertion and pore formation by a central region within TcdA and TcdB, denoted as translocation domain (TD) (Barth et al., 2001; Orrell et al., 2017). The glucosyltransferase domain (GTD) and the adjacent cysteine protease domain (CPD) are located at the N-terminus of both toxins (Jank and Aktories, 2008). The TD enables the translocation of the GTD and CPD across the endosomal membrane. Eventually, binding of cytosolic inositol hexakisphosphate to CPD triggers autocatalytic cleavage and release of the GTD into the cytosol (Giesemann et al., 2008; Egerer et al., 2009).

Earlier findings indicated that membrane cholesterol is crucial for the generation of translocation pores formed by TcdA and TcdB in endosomal membranes (Giesemann et al., 2006). More recently, we were able to show that an active sterol regulatory element–binding protein 2 (SREBP-2) pathway, which regulates cholesterol content in membranes, is required for efficient uptake of TcdA and TcdB into target cells (Papatheodorou et al., 2019). In addition, we demonstrated that the cholesterol-lowering drug simvastatin significantly reduces the intoxication of mouse embryonic fibroblasts (MEF cells) with TcdB *in vitro* (Papatheodorou et al., 2019).

Prompted by these observations and based on the recognized inhibitory role of the amphipathic steroid U18666A (3-β-[2-(diethylamino)ethoxy]androst-5-en-17-one) in cholesterol biosynthesis and/or intracellular transport (Cenedella, 2009), we investigated whether preincubation with the compound U18666A turns green monkey kidney epithelial cells (Vero), human cervix- (HeLa) and colon- (CaCo-2) cancer cells less prone to intoxication by TcdB. Importantly, U18666A exhibited a protective effect against TcdB in the tested cell lines. In addition, U18666A also protected cells from TcdA as well as from TcdA and TcdB from the epidemic NAP1/027 *C. difficile* strain, which is of particular clinical relevance.

Overall, the results corroborate the crucial role of membrane cholesterol for cell entry of TcdA and TcdB and should pave the way for the development of novel pharmacological antitoxin strategies for the supportive therapy after CDI.

## Materials and Methods

### Cell Culture

Minimum Essential Medium (MEM; Fisher Scientific GmbH, Schwerte, Germany; #11524426) supplemented with 10% fetal calf serum (FCS), 1% sodium pyruvate, 1% non-essential amino acids, and 1% penicillin/streptomycin was used for the cultivation of HeLa and Vero cells. CaCo-2 cells (CLS Cell Line Service GmbH, Eppelheim, Germany; #300137) were cultivated in Dulbecco’s Modified Eagle’s Medium (DMEM; Fisher Scientific GmbH, Schwerte, Germany; #11594486) supplemented with 10% FCS, 1% sodium pyruvate, 1% non-essential amino acids, and 1% penicillin/streptomycin. Alternatively, HeLa cells were grown in DMEM (Fisher Scientific GmbH, Schwerte, Germany; #13345364) supplemented with 10% FCS and 1% penicillin/streptomycin. Cells were maintained in the incubator under humidified conditions at 37°C and 5% CO_2_.

### Toxins and Reagents

TcdA from *C. difficile* VPI 10463 as well as TcdA and TcdB from a NAP1/027 strain were purified from 72 h culture supernatants using dialysis culture in Brain Heart Infusion broth as previously described (Lyerly et al., 1988; Pruitt et al., 2010). Toxin cross-contaminations were excluded by MALDI-TOF analysis. TcdB from *C. difficile* VPI 10463 was purified as described before (Just et al., 2008).

U18666A was ordered from Biomol (Hamburg, Germany; Cay10009085) or from Merck/Sigma-Aldrich (Darmstadt, Germany; U3633), dissolved in dimethyl sulfoxide (DMSO) to obtain a 20 mM stock solution and aliquoted prior to storage at −20°C.

If not otherwise stated, incubation of cells with *C. difficile* toxins and/or the U18666A compound occurred at 37°C.

### Microscopy

The following microscopes were used for monitoring toxin-induced cell rounding: Axiovert 40 CFL microscope (Carl Zeiss Microscopy, Jena, Germany) equipped with a ProgRes C10 plus camera (Jenoptik, Jena, Germany); Leica DMi1 equipped with a Leica MC170 HD camera (Leica, Wetzlar, Germany); Primovert (Carl Zeiss Microscopy, Jena, Germany); Lionheart FX Automated Microscope (BioTek, Vermont, United States). Toxin-induced cell rounding was manually quantified in microscopic images and, optionally, facilitated by the Neuralab online tool (https://neuralab.de).

### Preparation of Whole-Cell Lysates and Immunoblotting

Whole-cell lysates from cells growing in the wells from a 24-well plate were generated directly in wells by removing the growth medium and resuspending cell monolayers in 2.5-fold pre-heated Laemmli buffer (typically 30 μl per well). Lysate samples were heated at 95°C for 5 min, prior to SDS-PAGE and Western blotting of lysate proteins onto a nitrocellulose membrane. For the immunodetection of non-glucosylated Rac1 and GAPDH, primary mouse anti-Rac1 (clone 102; BD Biosciences, Heidelberg, Germany; #610651) and mouse anti-GAPDH (G-9; Santa Cruz Biotechnology, Dallas, United States; sc-365062) antibodies were used, respectively. Horseradish peroxidase (HRP)-coupled mouse IgG kappa binding protein (m-IgGκ BP-HRP; Santa Cruz Biotechnology, Dallas, United States; sc-516102) was used for developing antibody signals by the enhanced chemiluminescence (ECL) reaction.

### Measurement of Transepithelial Electrical Resistance

The EVOMX apparatus equipped with the STX2 electrode (World Precision Instruments, Sarasota, United States) was used for measuring the transepithelial electrical resistance (TEER) of CaCo-2 cells. At day 0, 1.2 × 10^5^ cells were seeded into 24-well hanging cell culture inserts (Brand GmbH, Wertheim, Germany; polyester membrane with pore size 0.4 μm) and incubated at 37°C until day 4. Optionally, at day 3, the U18666A compound was added basolaterally to the cells at a final concentration of 10 μM. Prior to TEER measurement at day 4, cell culture inserts were transferred into new wells containing only pre-warmed medium and further incubated at 37°C until TEER values (∼2,000 Ω) were stabilized. Then, TcdB (100 pM) was added apically to the cells and TEER measured every 30 min for up to 6 h. TEER values were normalized to time point 0 (t_0_, addition of the toxin), which was set to 100%.

### Statistics

Microsoft Excel (Student’s) *t*-test was used for calculating the significance of differences between mean values. Resulting *p*-values were indicated by asterisks as follows: *p < 0.05, ^**^p < 0.01, ^***^p < 0.001.

## Results

### U18666A Inhibits the Intoxication of HeLa Cells by TcdB

We initiated our study by using TcdB (derived from the historical *C. difficile* VPI 10463 strain) as representative for both *C. difficile* large glucosylating toxins and the human HeLa cervical carcinoma cell line as established model system for *in vitro* intoxication experiments. At first, we confirmed the biological activity of the prepared TcdB and identified an operable toxin concentration by analyzing cell rounding, which is a typical, specific, and well-established hallmark of intoxication of cells with TcdB or TcdA. Supplementary Figure 1A shows that TcdB (10 pM) directly added to HeLa cells lead to obvious cell rounding after 90 min of incubation and after 210 min, almost all cells were round. In contrast, without toxin treatment, the cells maintained their flat morphology (Supplementary Figure 1A). Thus, 10 pM of TcdB were used for the further experiments with HeLa cells.

U18666A is a pharmacological inhibitor of cholesterol biosynthesis and/or its intracellular transport (Cenedella, 2009). Therefore, a long-term incubation of cells with this compound is required to achieve a recognizable decrease in membrane cholesterol levels without causing cell toxicity. In this context, HeLa cells were incubated for 24 h with increasing concentrations of U18666A and their morphology was monitored microscopically for the same time period to identify the maximal tolerable concentration of U18666A. While HeLa cells tolerated U18666A concentrations up to 20 μM after overnight treatment (Supplementary Figure 1B), 50 μM U18666A was moderately toxic, as indicated by rounding up of a small proportion of cells, and 100 μM U18666A lead to complete disruption of the cell layer and detachment of the cells. Therefore, overnight treatment with up to 20 μM U18666A appears to be tolerable for HeLa cells. Nevertheless, we decided to limit the U18666A concentration at 10 μM in subsequent experiments.

**FIGURE 1:**
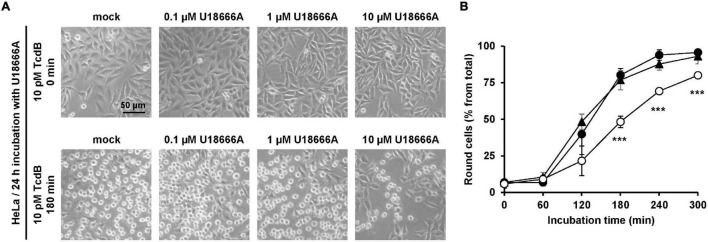
Effect of U18666A on intoxication of HeLa cells by TcdB. **(A)** HeLa cells were pretreated for 24 h with increasing concentrations of U18666A as indicated or were left untreated (mock), prior to addition of 10 pM TcdB. Microscopic images shown were taken after time point 0 min (upper row) and 180 min (lower row). **(B)** Quantification of the TcdB-induced cell rounding over time in mock-treated (black filled circles) and 24 h U18666A-pretreated HeLa cells (1 μM, black filled triangles; 10 μM, white filled circles). Graph shows mean (percentage values of round cells) from three experiments performed in parallel in independent wells. Error bars represent ± SD. Asterisks indicate statistical significance compared to control (mock-treated HeLa cells) with ***p < 0.001.

Next, we incubated HeLa cells with increasing concentrations of U18666A (0, 0.1, 1, and 10 μM) for 24 h, followed by the direct addition of 10 pM TcdB into the medium. Microscopic analysis of the cell morphology was performed at different time points after toxin application for up to 300 min. Figure 1A shows representative images at time points 0 and 180 min after TcdB addition. In direct comparison, preincubation of cells with 10 μM U18666A, but not with 0.1 μM or 1 μM U18666A, decreased the number of rounded cells in TcdB-treated cells (Figure 1A). Quantification of TcdB-induced cell rounding over time confirmed that HeLa cells pretreated with 10 μM U18666A were less affected by the toxin (∼25–30% reduced cell rounding after 180 min and 240 min), when compared to mock-pretreated cells and cells pretreated with 1 μM U18666A (Figure 1B).

### Incubation Time Impacts the U18666A-Mediated Inhibition of TcdB in HeLa Cells

**FIGURE 2:**
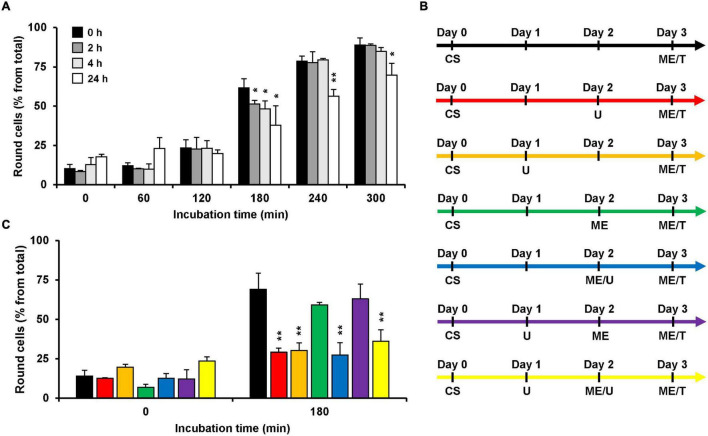
Effect of U18666A preincubation conditions on the TcdB intoxicaton of HeLa cells. **(A)** HeLa cells were preincubated for 0 h (black bars), 2 h (dark gray bars), 4 h (light gray bars), and 24 h (white bars) with U18666A, followed by TcdB intoxication (10 pM) and microscopic analysis of TcdB-induced cell rounding over time. The bar chart shows mean percentages of round cells at the indicated incubation times after addition of the toxin. Error bars represent ± SD. Asterisks indicate statistical significance at each time point compared to control (black bar) with *p < 0.05 and **p < 0.01. **(B,C)** The various U18666A pretreatment- and TcdB-intoxication conditions shown in **(C)** are schematically depicted with different colors in **(B)**. Cell seeding (CS) of HeLa cells at day 0 was followed by the addition of 10 μM U18666A (U) at day 1 (orange, purple and yellow) and/or at day 2 without (red) or with medium exchange (ME; blue, yellow) and TcdB intoxication (10 pM) by medium exchange (ME/T) at day 3. The bar chart in **(C)** shows the percentages of round cells at the indicated incubation times after addition of the toxin. Error bars represent ± SD. Asterisks indicate statistical significance at each time point compared to control (black bar for red and orange bar; green bar for blue, purple and yellow bar) with **p < 0.01. In all cases, data was obtained in three parallel experiments performed in independent wells.

Since U18666A interferes with the cholesterol biosynthesis and/or intracellular transport, the incubation time with the compound correlates with the extent of decrease in membrane cholesterol levels. Thus, we aimed to analyze the consequences of various incubation periods during pretreatment of HeLa cells with the U18666A compound on the subsequent intoxication of the cells by TcdB. To this end, we pretreated HeLa cells with 10 μM U18666A for 0, 2, 4, and 24 h, and exchanged the U18666A-containing medium with fresh medium including 10 pM TcdB. We then analyzed microscopically and quantified TcdB-induced cell rounding over time up to 300 min. In comparison to the cells without U18666A pretreatment, TcdB-induced cell rounding was inhibited only moderately (∼10% reduced cell rounding at time point 180 min) in cells pretreated for 2 and 4 h, respectively, and most prominent inhibition (∼25% reduced cell rounding at time point 180 min) was observed with a 24 h U18666A pretreatment (Figure 2A). Thus, comparatively short incubation periods of cells with the U18666A compound correlate with less efficient protection against TcdB intoxication. Since TcdB intoxication took place after removal of the U18666A compound by medium exchange, direct inhibitory effects of U18666A on the activity of the toxin in the cell culture supernatant can be excluded.

Next, we wanted to identify the best condition for the pretreatment of HeLa cells with U18666A, which are summarized schematically in Figure 2B, regarding the inhibition of TcdB intoxication in HeLa cells. For all conditions, cells were seeded into wells at day 0 and intoxicated at day 3 for 180 min with TcdB by exchanging the medium with fresh medium including 10 pM TcdB. In the control condition (Figure 2B, black arrow), cells were not pretreated with the U18666A inhibitor and cell rounding occurred in ∼70% of the cells (Figure 2C, black bar). Direct addition of 10 μM U18666A at day 2 (Figure 2B, red arrow; 24 h pretreatment) or day 1 (Figure 2B, orange arrow; 48 h pretreatment), reduced cell rounding equally to ∼30% (Figure 2C, red vs. orange bar). Thus, 48 h pretreatment of HeLa cells with U18666A was not superior to the 24 h pretreatment, in terms of TcdB inhibition. We also tested the sequential addition of 10 μM U18666A to cells, first by direct addition at day 1 and then by medium exchange at day 2 (Figure 2B, yellow arrow). Although cells were pretreated under this condition for a total of 48 h with the U18666A compound (but with U18666A renewal at day 2), TcdB inhibition was not superior to the 48 h pretreatment with U18666A without repeated application of the compound (Figure 2C, orange vs. yellow bar). Addition of 10 μM U18666A to cells at day 2 by medium exchange (Figure 2B, blue arrow) or by direct transfer into the wells (Figure 2B, red arrow), inhibited TcdB-induced cell rounding to equal extents (Figure 2C, red vs. blue bar). Interestingly, when U18666A (10 μM) was added to the cells at day 1 and removed again by medium exchange at day 2 (Figure 2B, purple arrow), TcdB-induced cell rounding was not inhibited and indistinguishable from the corresponding control without U18666A pretreatment at day 1 and medium exchange at day 2 (Figure 2C, green vs. purple bar). This finding suggests that membrane cholesterol levels recovered when U18666A was removed 24 h before toxin addition, either by cellular uptake from the medium or by biosynthesis.

### U18666A Confers Resistance Toward TcdB Intoxication in Vero Cells

**FIGURE 3:**
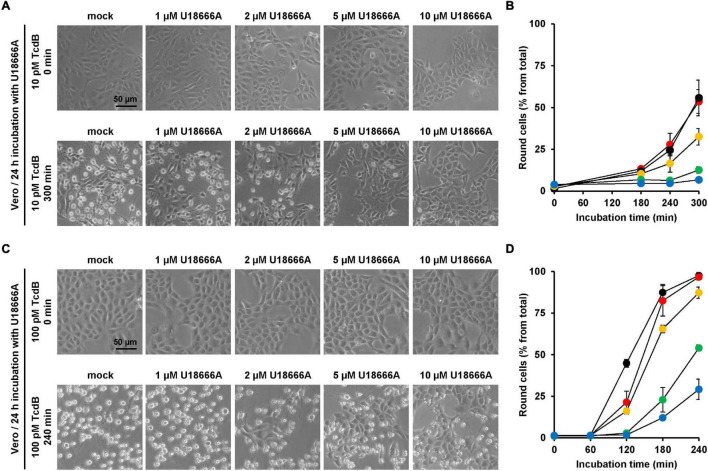
Effect of U18666A on the TcdB-induced cell rounding of Vero cells. **(A,C)** Vero cells pretreated for 24 h with increasing concentrations of U18666A as indicated or without pretreatment (mock) were intoxicated with **(A)** 10 pM or **(C)** 100 pM TcdB. Representative images were taken microscopically after time point 0 min [upper row in **(A,C)**] and 300 min [lower row in **(A)**] or 240 min [lower row in **(C)**]. **(B,D)** Quantification of the TcdB-induced cell rounding over time in mock-treated (black filled circles) and 24 h U18666A-pretreated HeLa cells (1 μM, red filled circles; 2 μM, orange filled circles; 5 μM, green filled circles, 10 μM, blue filled circles). Graphs in **(B,D)** show mean percentage values of round cells as indicated by three parallel experiments performed in independent wells. Error bars represent ± SD.

Next, the protective effect of U18666A against TcdB intoxication in a second cell line was investigated. African green monkey kidney (Vero) cells were used. Due to their flat morphology, Vero cells are a well-established model cell line for studying TcdB effects. Vero cells were pretreated for 24 h with increasing U18666A concentrations (0, 1, 2, 5, 10 μM) and, subsequently, TcdB was added to the cells at a final concentration of 10 and 100 pM, respectively. TcdB-induced cell rounding was analyzed microscopically for up to 300 min (10 pM TcdB) or 240 min (100 pM TcdB). Figures 3A,C show representative images of mock- and U18666A-pretreated Vero cells at time points zero and 300 min (10 pM TcdB) and at time points zero and 240 min (100 pM), respectively, after toxin addition. Diagrams in Figures 3B,D show the corresponding quantification of TcdB-induced cell rounding over time for 10 pM or 100 pM TcdB, respectively. After 300 min, cell rounding induced by 10 pM TcdB was strongly reduced to ∼10% in Vero cells pretreated with 5 and 10 μM U18666A vs. mock-pretreated cells (∼60% cell rounding) (Figure 3B). In cells intoxicated with 100 pM TcdB, nearly 100% cell rounding was observed after 210 min, which was decreased to ∼50% by pretreatment with 5 μM U18666A and to ∼25% by pretreatment with 10 μM U18666A. Thus, U18666A confers robust resistance in Vero cells toward 10 pM TcdB and moderate resistance toward 100 pM TcdB. Notably, the protective effect of U18666A against TcdB was more pronounced in Vero than in HeLa cells.

**FIGURE 4:**
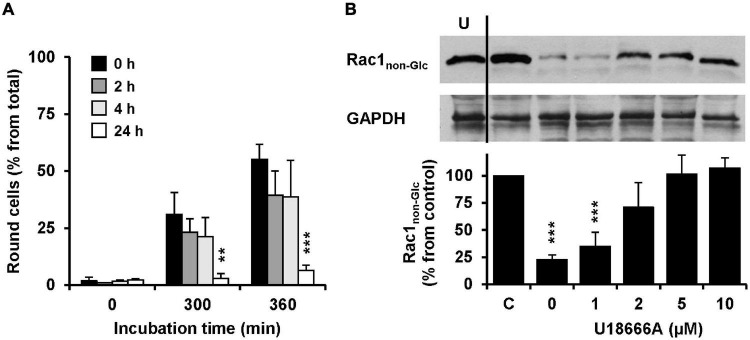
Effect of U18666A on the TcdB-induced Rac1 glucosylation and role of U18666A pretreatment duration on TcdB-induced cell rounding in Vero cells. **(A)** Vero cells were preincubated for 0 h (black bars), 2 h (dark gray bars), 4 h (light gray bars), and 24 h (white bars) with the U18666A compound, prior to TcdB intoxication (10 pM) and microscopic analysis of TcdB-induced cell rounding over time. The mean percentage values of round cells at the indicated incubation times after addition of the toxin and from three parallel experiments performed in independent wells are plotted in the bar chart. Error bars represent ± SD. Asterisks indicate statistical significance at each time point compared to control (black bar) with **p < 0.01 and ***p < 0.001. **(B)** Immunoblots against non-glucosylated Rac1 (Rac1_non–Glc_) and GAPDH (loading control) are shown (upper panel), obtained with whole-cell lysates that were generated from Vero cells pretreated for 24 h with increasing concentrations of U18666A as indicated and after intoxication for 120 min with 100 pM TcdB. In a control sample (C) cells were left without U18666A pretreatment and toxin addition. In another control (U) cells were preincubated with 10 μM U18666A but were left without toxin treatment. The bar chart (lower panel) shows the quantification of the Rac1 signals (normalized with the signals of the GAPDH loading control) relative to the control sample (C), which was set to 100%. Error bars represent ± SEM, calculated from six experiments performed in independent wells (three experiments in parallel on two different days). Asterisks indicate statistical significance at each time point compared to control (C) with ***p < 0.001.

The influence of the pretreatment time of U18666A on the intoxication of Vero cells with TcdB was analyzed. To this end, Vero cells were pretreated with 10 μM U18666A for 0, 2, 4, and 24 h, prior to intoxication with 10 pM TcdB. Eventually, TcdB-induced cell rounding was analyzed microscopically and quantified after 0, 300, and 360 min. Importantly, 24 h pretreatment with the U18666A compound strongly inhibited TcdB-induced cell rounding (from ∼60% in mock-pretreated cells to ∼10% after 360 min of TcdB intoxication) (Figure 4A, black bar vs. white bar). Pretreatment with 10 μM U18666A for 2 and 4 h did not reduce TcdB-induced cell rounding significantly after 300 and 360 min of intoxication, when compared to mock-pretreated cells (Figure 4A, black bar vs. dark gray bar and black bar vs. light gray bar). Thus, also in Vero cells the incubation time has a major impact on the U18666A-mediated inhibition of TcdB. Similarly as observed with HeLa cells, a short incubation of Vero cells with the U18666A compound inefficiently protects these cells against TcdB intoxication.

To confirm our findings with another experimental approach, we generated whole-cell lysates from Vero cells intoxicated for 120 min with 100 pM TcdB, followed by immunoblotting against the TcdB target protein Rac1 with an antibody that detects only the non-glucosylated form of Rac1. Prior to TcdB intoxication the cells were pretreated for 24 h with increasing concentrations of U18666A (0, 1, 2, 5, and 10 μM). Whole-cell lysates obtained from untreated cells were used as control for maximal non-glucosylated Rac1 signal in the immunoblot. As expected, the Rac1 signal in whole-cell lysates obtained from TcdB-intoxicated cells without U18666A pretreatment was decreased to ∼10–20% from control (100%) (Figure 4B). However, the Rac1 signal was present almost to the level of the control in TcdB-intoxicated cells pretreated either with 5 or 10 μM U18666A. Lower U18666A concentrations (1 or 2 μM) protected cells less effectively toward TcdB intoxication, because the Rac1 signal was found to be reduced to ∼30% (1 μM U18666A) and ∼70% (2 μM U18666A), respectively, in these samples. Notably, pretreatment of cells with U18666A had no influence on the Rac1 expression level or on the binding by the glucosylation-sensitive anti-Rac1 antibody.

### U18666A Decreases the Sensitivity of Human Intestinal Epithelial CaCo-2 Cells Against TcdB

**FIGURE 5:**
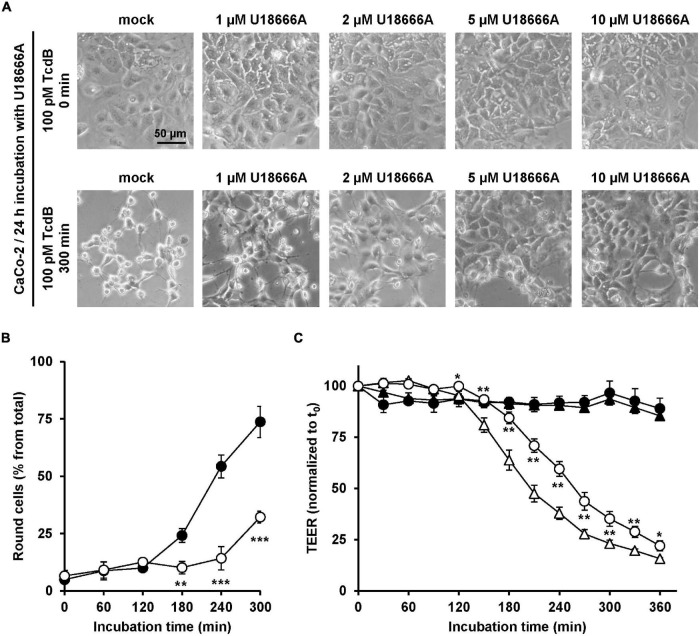
Effect of U18666A on the intoxication of CaCo-2 cells by TcdB. **(A)** After pretreatment of CaCo-2 cells for 24 h with increasing concentrations of U18666A as indicated or without pretreatment (mock), cells were intoxicated with 100 pM TcdB for 300 min and cell morphology analyzed microscopically. Representative images after time point 0 min (upper row) and 300 min (lower row) are shown. **(B)** Quantification of the TcdB-induced cell rounding over time in mock-treated (black filled circles) and 24 h U18666A (10 μM)-pretreated CaCo-2 cells (white filled circles). Graph shows mean values (percentage of round cells) from three parallel experiments performed in independent wells. Error bars represent ± SD. Asterisks indicate statistical significance compared to control (mock-treated CaCo-2 cells) with **p < 0.01 and ***p < 0.001. **(C)** CaCo-2 cells grown in inserts were pretreated for 24 h with 10 μM U18666A (circles) or were left without pretreatment (triangles), prior to apical addition of 100 pM TcdB (white filled circles and triangles) at time point 0 (t_0_) and measurement of the transepithelial electrical resistance (TEER) at indicated incubation times. Parallel samples were not treated with TcdB (black filled circles and triangles). Diagram shows relative TEER values normalized to time point 0 (t_0_). Error bars represent ± SD. Asterisks indicate statistical significance at each time point between white filled triangles (–U18666A/+TcdB) and white filled circles (+U18666A/+TcdB), with *p < 0.05 and **p < 0.01.

Since *C. difficile* is a pathogen of the human intestine, it was relevant to test whether U18666A confers resistance toward TcdB intoxication in the medically and physiologically more relevant human colon carcinoma cell line CaCo-2. To this end, CaCo-2 cells were preincubated for 24 h with increasing concentrations of U18666A (0, 1, 2, 5, and 20 μM), prior to addition of 100 pM TcdB and subsequent microscopic analysis of the cell morphology over time. Pretreatment of CaCo-2 cells with increasing U18666A concentrations correlated with less microscopically observable cell rounding after incubation with TcdB for 300 min, when compared to mock-pretreated cells (Figure 5A). The quantification of TcdB-induced cell rounding over time of mock- and 10 μM U18666A-pretreated cells confirmed the protective effect of U18666A against TcdB also in CaCo-2 cells (Figure 5B). Pretreatment of CaCo-2 cells with 10 μM U18666A decreased TcdB-induced cell rounding to ∼25% after 300 min of intoxication.

To investigate the protective effect of U18666A on the intoxication of CaCo-2 cells by TcdB by a further end point, the integrity of the barrier of function of CaCo-2 cells, grown as polarized monolayers in hanging cell culture inserts, was analyzed in terms of the transepithelial electrical resistance (TEER). Accordingly, CaCo-2 cell monolayers were pretreated with 10 μM U18666A for 24 h or were left untreated (mock) before apical addition of 100 pM TcdB. Then, TEER was measured every 30 min over a period of 6 h after intoxication. In direct comparison, the TcdB-induced TEER decrease over time was delayed in U18666A-pretreated CaCo-2 cells when compared to mock-pretreated cells (Figure 5C). Thus, U18666A pretreatment reduced the TcdB-induced disruption of the epithelial integrity of CaCo-2 cells.

### U18666A Confers Protection Against VPI 10463-Derived TcdA and NAP1/027-Derived TcdA and TcdB

**FIGURE 6:**
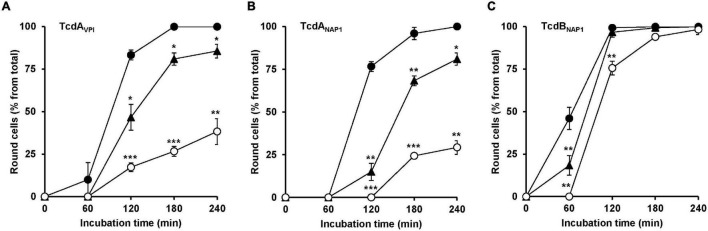
Effect of U18666A on the intoxication of HeLa cells by TcdA_VPI_, TcdA_NAP1_ and TcdB_NAP1_. HeLa cells preincubated with increasing concentrations of U18666A (0 μM, black filled circles; 1.25 μM, black filled triangles and 10 μM, white filled circles) were intoxicated with **(A)** 10 pM VPI-derived TcdA (TcdA_VPI_), **(B)** 10 pM NAP1/027-derived TcdA (TcdA_NAP1_) or **(C)** 1 pM NAP1/027-derived TcdB (TcdB_NAP1_), prior to microscopic analysis of the cell morphology over time. Graphs in **(A–C)** show means (percentage values of round cells) from three experiments performed in parallel in independent wells. Error bars represent ± SD. Asterisks indicate statistical significance compared to control (mock-treated HeLa cells) with *p < 0.05, **p < 0.01, and ***p < 0.001.

Finally, it was tested whether U18666A also protects cells against TcdA from the historical *C. difficile* strain VPI 10463 and against TcdA and TcdB from the epidemic *C. difficile* strain NAP/027. For that purpose, HeLa cells were pretreated for 24 h with increasing concentrations of U18666A (0, 1.25, and 10 μM), prior to addition of 10 pM VPI-derived TcdA (TcdA_VPI_), 10 pM NAP1/027-derived TcdA (TcdA_NAP1_) or 1 pM NAP1/027-derived TcdB (TcdB_NAP1_). Subsequently, microscopic analysis of the cell morphology was performed. As summarized in Figure 6, a 24 h pretreatment of HeLa cells with 10 μM U18666A effectively diminished cell rounding induced by TcdA_VPI_ (Figure 6A), TcdA_NAP1_ (Figure 6B) or TcdB_NAP1_ (Figure 6C). It is of note, however, that the reduction of TcdB_NAP1_ intoxication by U18666A was less effective when compared to TcdA_VPI_ and TcdA_NAP1_, respectively, both of which were inhibited to an equal extent by the U18666A pretreatment.

## Discussion

The crucial role of membrane cholesterol for pore formation of TcdA and TcdB during their endocytic uptake into target cells was first described by the Aktories group in 2006 (Giesemann et al., 2006). Along with this finding, we found more recently that an active and functional SREBP-2 pathway, which plays a major role in cholesterol metabolism, is required in target cells for the cytotoxicity of TcdA and TcdB and showed that the hypocholesterolemic drug simvastatin, which inhibits the HMG-CoA reductase, a key enzyme in cholesterol biosynthesis, efficiently protected cultured mammalian cells from TcdB intoxication (Papatheodorou et al., 2019). Since there is no structure of membrane-embedded TcdA or TcdB, the exact interaction between membrane cholesterol and TcdA/TcdB is not known. However, a recently discovered “mirror code” controlling protein-cholesterol interactions in the outer and inner membrane leaflets (Fantini et al., 2016) seems to be present also within a region of TcdA and TcdB (amino acids 830–990) (Gerhard, 2017), which is in charge of pore formation (Genisyuerek et al., 2011). Cholesterol might be crucial for the insertion of the translocation domain of the toxins into the endosomal membrane. Otherwise, membrane cholesterol could serve as a platform for the correct positioning of transmembrane segments of the toxins within the endosomal membrane for generating a translocation-competent pore.

Prompted by the above mentioned observations that membrane cholesterol is crucial for the uptake of TcdA and TcdB in target cells, the U18666A compound came into our focus, since it has been described by several studies as potent inhibitor of cholesterol biosynthesis and/or intracellular trafficking (Cenedella, 2009). At least three membrane-bound enzymes in the cholesterol biosynthetic pathway, namely 2,3-oxidosqualene cyclase, desmosterol reductase and cholestenol delta-isomerase, are known to be inhibited by the U18666A compound (Duriatti et al., 1985; Bae and Paik, 1997; Moebius et al., 1998). The activity of HMG-CoA reductase, another key enzyme in cholesterol biosynthesis, is decreased by addition of low concentrations of U18666A, but increased at higher drug levels (Panini et al., 1984). Another feature of U18666A is the inhibition of the intracellular transport of cholesterol, namely from the lysosome to the endoplasmic reticulum (ER) and to the plasma membrane (PM), respectively, and from the PM to the ER (Liscum and Dahl, 1992; Underwood et al., 1998). Effects of U18666A on intracellular cholesterol trafficking are explained by its direct interference with the lysosomal membrane protein Niemann-Pick type C1 (NPC1), which transports cholesterol from receptor-mediated uptake of low-density lipoproteins (LDL) to post-lysosomal destinations (Carstea et al., 1997; Blanchette-Mackie, 2000; Lange et al., 2000; Lu et al., 2015).

The present study revealed that the U18666A compound confers increased protection to various mammalian cell lines against the *C. difficile* toxin TcdB. In addition, TcdA-inhibition by U18666A-pretreatment was confirmed in HeLa cells and TcdA-inhibition by U18666A was more prominent when compared to TcdB, irrespective of the source strain of both toxins. Inhibition of TcdB is more challenging most likely due to the fact that TcdB preparations are up to 1,000-fold more toxic than TcdA preparations (Rothman et al., 1984). It is also feasible that TcdA membrane insertion and/or pore formation in endosomal membranes is more prone to changes of membrane cholesterol levels. Moreover, pretreatment with the U18666A compound was not equally protective against TcdB among the various tested cells. For instance, the inhibition of TcdB-intoxication by U18666A pretreatment was more prominent in Vero cells when compared to HeLa cells at equal inhibitor concentrations. Possible explanations for this observation could be that either the compound is more efficiently taken up into Vero cells or that the compound is more efficiently degraded or excreted from HeLa cells by drug transporters. It is also feasible that membrane cholesterol levels and/or cholesterol uptake, intracellular transport and biosynthesis capabilities are vastly differing between HeLa and Vero cells, thus influencing the inhibitory potential of the U18666A compound in each cell line.

The replication of many viruses is associated with intact cholesterol biosynthesis and intracellular transport. Thus, it has been reported that U18666A suppress the replication of several viruses, such as ebola virus, dengue virus, human hepatitis C virus, and type I feline coronavirus (Poh et al., 2012; Lu et al., 2015; Elgner et al., 2016; Takano et al., 2017). The mechanism underlying the U18666A-mediated inhibition of TcdB and TcdA is likely related to a blockage of cholesterol biosynthetic enzymes and of the intracellular transport of endocytosed cholesterol from the culture medium to the plasma membrane and/or to endocytic vesicles. Consistent with this hypothesis, cells were strongly protected from toxin intoxication, when they were pretreated for at least 24 h with U18666A, but not after shorter incubation periods with the compound. We widely excluded the possibilities of (i) an inactivating effect of U18666A by direct binding to the toxins or (ii) through alteration of endosomal membranes through U18666A incorporation, because there was no efficient protection from TcdB after short incubation periods with U18666A, but protection when the U18666A-containing medium on top of cells was removed by replacement with TcdB-containing medium during the intoxication step.

The cholesterol-dependent pore formation by TcdA or TcdB in endosomal membranes is a crucial step during cell entry of both toxins and this study corroborated that this specific step can be targeted by pharmacological tools such as U18666A. Importantly, U18666A also inhibited TcdA and TcdB variants from the epidemic NAP1/027 *C. difficile* strain. This finding indicates that cholesterol seems to be a conserved player in TcdA/TcdB intoxication. Thus, the development of cholesterol-lowering drugs, such as U18666A, as potential therapeutics against TcdA and TcdB might be of interest not only for the treatment of infections caused by non-epidemic *C. difficile* strains (such as VPI 10463), but also for the treatment of diseases associated with infections caused by the epidemic NAP1/027 *C. difficile* strain. However, the safety profile of U18666A for clinical use is not evaluated so far yet. Cataracts were described in rats that were treated daily with 10 mg/kg U18666A, but not when the rats were treated every fourth day with the U18666A compound (Cenedella and Bierkamper, 1979). Thus, the drug application form (enteral or rectal administration) and interval should be carefully considered in potential clinical studies on the efficacy and safety of U18666A and derivatives in the (supportive) treatment of severe CDI.

## Data Availability Statement

The raw data supporting the conclusions of this article will be made available by the authors, without undue reservation.

## Author Contributions

PP, SF, EC-O, and HB designed the research. PP, SK, AB-L, SF, ED, TT, AW, and SS performed the research. PP, SK, AB-L, SF, KA, EC-O, CR, and HB analyzed the data. PP, KA, CR, and HB wrote the manuscript. All authors contributed to the article and approved the submitted version.

## Conflict of Interest

The authors declare that the research was conducted in the absence of any commercial or financial relationships that could be construed as a potential conflict of interest.

## Publisher’s Note

All claims expressed in this article are solely those of the authors and do not necessarily represent those of their affiliated organizations, or those of the publisher, the editors and the reviewers. Any product that may be evaluated in this article, or claim that may be made by its manufacturer, is not guaranteed or endorsed by the publisher.
