# Supplementary material for: The Compound U18666A Inhibits the Intoxication of Cells by Clostridioides difficile Toxins TcdA and TcdB
Source: Front Microbiol. 2021 Nov 29;12:784856. doi: 10.3389/fmicb.2021.784856 (PMC8667575; doi:10.3389/fmicb.2021.784856)
Supplement: Supplementary file 1 [file Data_Sheet_1.PDF]

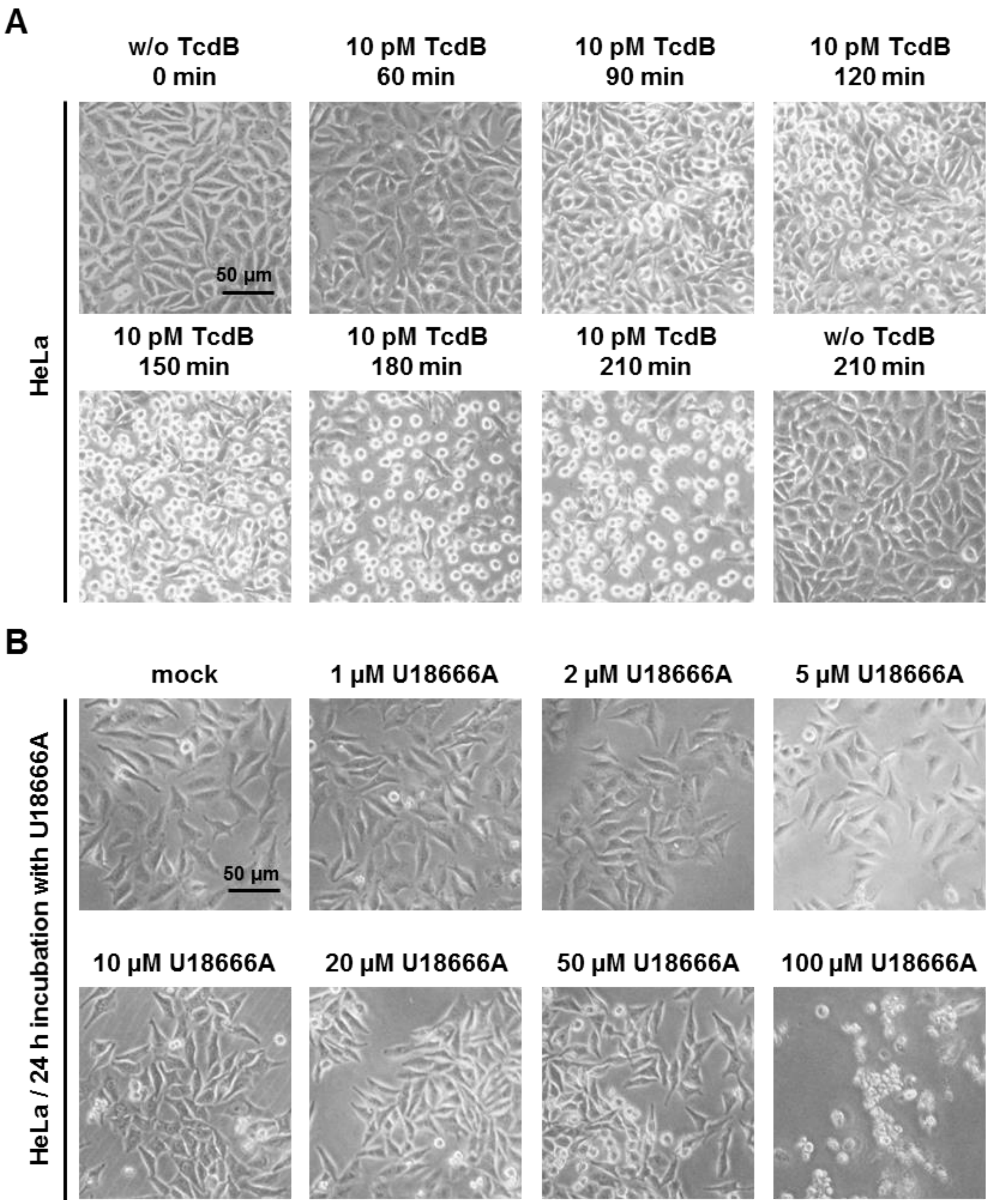

Supplementary Figure 1. Effect of TcdB and U18666A on HeLa cells. (A) HeLa cells grown in culture wells were (A) intoxicated with 10 pM TcdB, followed by the microscopic analysis of the cell morphology at the indicated time points or (B) incubated for 24 h with increasing concentrations of U18666A as indicated, followed by the microscopic analysis of the cell morphology. Cells without TcdB (w/o TcdB) in (A) or without U18666A pretreatment (mock) in (B) were used as negative controls.
